# Bacterial Community Dynamics and Biocement Formation during Stimulation and Augmentation: Implications for Soil Consolidation

**DOI:** 10.3389/fmicb.2017.01267

**Published:** 2017-07-11

**Authors:** Navdeep K. Dhami, Walaa R. Alsubhi, Elizabeth Watkin, Abhijit Mukherjee

**Affiliations:** ^1^Biologically Activated Materials Laboratory, Department of Civil Engineering, Curtin University Perth, WA, Australia; ^2^School of Biomedical Sciences, Curtin Health Innovation Research Institute-Biosciences, Curtin University Perth, WA, Australia

**Keywords:** microbial carbonate precipitation, biostimulation, bioaugmentation, urease, carbonic anhydrase

## Abstract

Microbially-induced CaCO_3_ precipitation (MICP) is a naturally occurring process wherein durable carbonates are formed as a result of microbial metabolic activities. In recent years, MICP technology has been widely harnessed for applications in civil engineering wherein synthesis of calcium carbonate crystals occurs at ambient temperature paving way for low energy biocement. MICP using pure urease (UA) and carbonic anhydrase (CA) producing bacteria has been promising in laboratory conditions. In the current study we enriched ureolytic and carbonic anhydrase communities in calcareous soil under biostimulation and bioaugmentation conditions and investigated the effect of microbial dynamics on carbonate precipitation, calcium carbonate polymorph selection and consolidation of biological sand column under nutrient limited and rich conditions. All treatments for stimulation and augmentation led to significant changes in the composition of indigenous bacterial population. Biostimulation as well as augmentation through the UA route was found to be faster and more effective compared to the CA route in terms of extracellular enzyme production and carbonate precipitation. Synergistic role of augmented cultures along with indigenous communities was recorded via both the routes of UA and CA as more effective calcification was seen in case of augmentation compared to stimulation. The survival of supplemented isolates in presence of indigenous bacterial communities was confirmed through sequencing of total diversity and it was seen that both UA and CA isolate had the potential to survive along with native communities under high nutrient conditions. Nutrient conditions played significant role in determining calcium carbonate polymorph fate as calcitic crystals dominated under high carbon supplementation. Finally, the consolidation of sand columns via stimulation and augmentation was successfully achieved through both UA and CA route under high nutrient conditions but higher consolidation in short time period was noticed in UA route. The study reports that based upon the organic carbon content in native soils, stimulation can be favored at sites with high organic carbon content while augmentation with repeated injections of nutrients can be applied on poor nutrient soils via different enrichment routes of microbial metabolism.

## Introduction

Microbial activities in a variety of environments have influenced the formation of geological formations as microbialites, aquifers, cave speleothems, sediments, mats, rocks (Dupraz, C. et al., 2009; Rusznyak et al., 2012; Zhu and Dittrich, 2016). Their diverse metabolic pathways including photosynthesis, ureolysis, ammonification, denitrification, methane oxidation, ammonification in addition to extracellular polymeric substances have been reported to influence redox conditions and are responsible for inducing calcium carbonate precipitation in a range of natural systems (Braissant et al., 2007; Dupraz, S. et al., 2009). Recent awareness in the application of such cementing bacteria in formation of calcium carbonate binders in *in vitro* conditions has led to emergence of Microbially induced carbonate precipitation (MICP) technology for several engineering problems as durable carbonates can be synthesized at ambient temperatures by utilizing certain classes of bacteria (Zhu and Dittrich, 2016). The applications of this technology are widespread from metal remediation, oil recovery, CO_2_ sequestration to remediation and restoration of construction materials (De Muynck et al., 2010; Dejong et al., 2013; Dhami et al., 2014a). As this technology offers the benefit of high sustainability over conventional methods due to its synthesis at ambient temperature, it is becoming widely acceptable tool for synthesis of bacterial based cements. Especially in case of soils, the ubiquity of bacteria (around 10^12^ microbes per kg of soil) is seen as a resource for *in situ* cementation applications in soil strengthening (De Muynck et al., 2010; Dejong et al., 2013; Dhami et al., 2016b).

The basic process of carbonate production in nature is governed by four key factors: (1) the calcium concentration, (2) the concentration of dissolved inorganic carbon (DIC), (3) the pH, and (4) the availability of nucleation sites (Hammes et al., 2003). Microorganisms, which act as sites of nucleation can influence the carbonate precipitation by altering almost any of the precipitation parameters described, either separately or in combination with one another (Hammes and Verstraete, 2002). Different classes of both autotrophic and heterotrophic bacteria as cyanobacteria, ureolytic bacteria, nitrate reducing bacteria, myxobacteria, sulfate reducing bacteria, methanogenic bacteria play a role in inducing calcium carbonate precipitation in natural formations either through an increase in pH or through DIC (Dhami et al., 2013c; Zhu and Dittrich, 2016). Amongst these, ureolysis and photosynthetic metabolism has been found to be dominating in most of terrestrial systems while in case of fresh water and marine environments, photosynthesis along with sulfate reduction pathway have been reported to be dominating. A few studies demonstrated the active role of microbes in carbonate mineralization in natural structures and also investigated microbial diversity associated with these formations as caves, stromatolites, sediments (Dupraz, C. et al., 2009; Dupraz, S. et al., 2009; Banks et al., 2010; Rooney et al., 2010; Rusznyak et al., 2012). In case of caves, bacterial phyla Proteobacteria, Actinobacteria and Acidobacteria were found to be dominant groups in a number of studies (Bastian et al., 2009; Wu et al., 2015; Zepeda Mendoza et al., 2016) while in case of stromatolites, photosynthetic microorganisms as cyanobacteria have been implicated for formation of carbonates in the photic zones (Steneck et al., 1998; Spadafora et al., 2010; Bosak et al., 2013). Recently Gleeson et al. (2016) and Suosaari et al. (2016) found the prevalence of Proteobacteria, Bacteroidetes, Cyanobacteria and Firmicutes phylum in stromatolites.

Amongst the different bacterial metabolic pathways described above, ureolytic and carbonic anhydrase routes have been explored widely for civil engineering applications of carbonates (Smith and Ferry, 2000; De Muynck et al., 2010; Qian et al., 2015; Dhami et al., 2016a). Through urea hydrolysis, ureolytic communities impact the concentration of DIC and pH. In this process, urease enzyme hydrolyses urea to produce carbonate (1) which hydrolyses spontaneously to ammonia and carbonic acid (2). These products equilibrate to form bicarbonate in the presence of water wherein ammonium and hydroxide ions are formed leading to pH increase (3,4). In the presence of calcium and alkaline environment, these reactions pave the way to calcium carbonate precipitation (5) (Stocks-Fischer et al., 1999).

(1) $$\begin{array}{rcl} \text{CO }{\left({\text{NH}}_{2}\right)}_{2}+{\text{H}}_{2}\text{O} & \overset{\text{Urease}}{\to } & {\text{NH}}_{2}\text{COOH}+{\text{NH}}_{3} \end{array}$$

(2) $$\begin{array}{rcl} {\text{NH}}_{2}\text{COOH}+{\text{H}}_{2}\text{O} & \to & {\text{NH}}_{3}+{\text{H}}_{2}{\text{CO}}_{3} \end{array}$$

(3) $$\begin{array}{rcl} {\text{H}}_{2}{\text{CO}}_{3} & \to & 2{\text{H}}^{+}+{\text{CO}}_{3}^{2-} \end{array}$$

(4) $$\begin{array}{rcl} {\text{NH}}_{3}+{\text{H}}_{2}\text{O} & \to & {\text{NH}}^{4+}+{\text{OH}}^{-} \end{array}$$

(5) $$\begin{array}{rcl} {\text{Ca}}^{2+}+{\text{CO}}_{3}^{2-} & \to & {\text{CaCO}}_{3} \end{array}$$

The prevalence of ureolytic communities has been widely seen in different soils irrespective of the type, mineralogy, environmental conditions (Fujita, 2008; Gat et al., 2016; Zhu and Dittrich, 2016). Another enzyme Carbonic anhydrase (CA) has also been demonstrated to be ubiquitously distributed and play a significant role in capturing CO_2_ in the form of carbonates in nature (Tripp et al., 2001; Dhami et al., 2014b, 2016a). This enzyme acts as a potential biological catalyst for hydration of CO_2_ which in the presence of a calcium source leads to production of calcium carbonate (6, 7):

(6) $$\begin{array}{r} {\text{CO}}_{2}+{\text{H}}_{2}\text{O}\overset{\text{CA}}{↔}{\text{H}}_{2}{\text{CO}}_{3}↔{\text{HCO}}_{3}^{-}+{\text{H}}^{+} \end{array}$$

(7) $$\begin{array}{rcl} {\text{Ca}}^{2+}+2{\text{HCO}}_{3}^{-}\to {\text{CaCO}}_{3}+{\text{HCO}}_{3}^{-} & + & {\text{H}}^{+}+\to {\text{CaCO}}_{3}\\ + & {\text{CO}}_{2}+{\text{H}}_{2}\text{O} \end{array}$$

Not much has been reported on prevalence of bicarbonate utilizing carbonic anhydrase communities in soils. Recently Srivastava et al. (2015) isolated several carbonic anhydrase bacterial isolates after enrichment from marble rock soils which had the potential to utilize bicarbonate ions as sole source of carbon. Successful generation of carbonate utilizing bacterial carbonic anhydrase has been found through supplementation of CO_2_ as well as NaHCO_3_ (Sharma and Bhattacharya, 2010; Dhami et al., 2014b; Kaur et al., 2016).

In case of soil applications, there are generally two approaches for MICP: biostimulation and bioaugmentation. In the case of biostimulation, the natural indigenous microbes are stimulated/enriched by the addition of specific nutrients and carbon sources to promote specific class of microbes (Gat et al., 2016). It relies on the ubiquity of calcifying bacteria in those soils as well as their spatial distribution. This method faces the challenge of long time periods required for effective output. In the case of bioaugmentation, the system is supplemented with exogenous bacteria. The potential of foreign cultures to survive and work effectively in a new environment is challenging due to competition from native communities which affect their survival as well as their metabolic potential along encountering of poor compatibility in new environment (Wenderoth et al., 2003; Baek et al., 2007; Gat et al., 2016).

With relation to MICP, only a few studies have been conducted on biostimulation in indigenous soils for cementation and there also, the efficacy of the process was monitored in terms of carbonate precipitation only (Fujita, 2008; van Paassen et al., 2010). In one of the recent studies of Gat et al. (2016), microbial dynamics were studied under ureolytic enrichment and prevalence of Firmicutes was reported which also contributed to significant ureolysis. Similarly in case of carbonic anhydrase, Ueda et al. (2012) reported that active carbonic anhydrases in natural environments are associated with Proteobacteria. But no reports are available on microbial dynamics and their calcium carbonate precipitation potential following stimulation of ureolytic and carbonic anhydrase routes for biocementation applications. Also in case of Bioaugmentation, pure cultures of *Sporosarcina pasteurii* (UA) and *Bacillus* sp. (CA) have been reported to be successful in production of carbonate precipitation in laboratory conditions with sterile soils but not much is available on potential of these cultures in presence of indigenous communities in non-sterile environments of fields (Ivanov and Chu, 2008; Wei et al., 2011; Dejong et al., 2013; Dhami et al., 2013a; Khun, 2014). Whether stimulation can be a viable approach for microbial calcification using different metabolic routes of UA and CA and whether the augmented bacterial cultures compete with indigenous communities under different enrichment conditions needs to be explored along with the potential of calcification. So, we aim to analyse microbial dynamics and metabolic activities under different enrichment conditions (stimulation and augmentation) for ureolytic and carbonic anhydrase communities in relation to their calcification potential. As the availability of nutrients in different soils is a crucial factor for microbial growth and metabolism, there is need to investigate the effect of different concentrations of organic carbon supplementation on microbial metabolism and calcification potential.

Another parameter that influences the efficacy of MICP for soil cementation is the carbonates polymorph. Calcium carbonates exist in different polymorphs with varying morphologies and characteristics as calcite (rhombic), aragonite (needle like), vaterite (spherical), two hydrated crystalline phases monohydrocalcite, ikaite and amorphous phases (Rodriguez-Navarro et al., 2012; Ronholm et al., 2014; Bains et al., 2015; Anbu et al., 2016). The formation of these polymorphs has been reported to be influenced by a number of parameters including bacterial species involved and metabolic pathway associated as they control the amount of dissolved organic carbon, which is the major player in polymorph selection (Rodriguez-Navarro et al., 2012; Dhami et al., 2013b). The fate of carbonate polymorph further affects the efficacy of MICP as few polymorphs are more stable and durable compared to others as calcite has been recorded to have higher mechanical strength properties compared to other polymorphs (Dhami et al., 2016a). In order to test the potential of MICP via different routes, it is also paramount to investigate the fate of carbonate polymorphs formed as they play important role in determining the efficacy of biocementation.

With this background, we aimed to explore the effect of biostimulation and bioaugmentation through ureolytic and carbonic anhydrase route on (a) microbial community dynamics and metabolism, (b) calcium carbonate precipitation efficacy, (c) carbonate polymorph synthesis.

## Methods

### Microorganisms used in the study

Ureolytic bacteria *Sporosarcina pasteurii ATCC 11859 (UB)* was obtained from *in vitro* Technologies Australia and Carbonic anhydrase producing bacterial culture *Bacillus cereus C1* (CB) isolated from speleothems of Margaret river caves, Western Australia, Australia were used as augmented cultures.

### Culturing conditions for stimulation and augmentation in flasks

Calcareous soil samples were collected from the Margaret River region of Western Australia, Australia. These soils are classified as alkaline and calcareous as per Schoknecht and Pathan (2013). The soils were collected from a depth of 0.2 m using sterile auger (washed with 70% ethanol) and stored at 4°C till further analysis. The characteristics of the soil are listed in Table 1.

**Table 1:** Properties of soil used for current study.

**Parameter**	**Value**
pH	8.5 ± 0.2
Conductivity	2.1 mS/cm
D_50_	0.37 mm
Moisture content	7%
Organic carbon	1–2%

The collected sand was suspended in Artificial ground water (AGW) media as described in Gat et al. (2016) with different supplements based upon the enrichment treatment type, i.e., biostimulation or bioaugmentation via Ureolytic route and Carbonic anhydrase route under high and low nutrient conditions.

Briefly, 10 g of sand (in triplicates) was suspended in 100 ml of each of the sterile media mentioned in Table 2 (prepared by autoclaving). Urea, CaCl_2_, NaHCO_3_, NiCl_2_, and ZnSO_4_ were added after filter sterilization into individual flasks (Nickel and Zinc act as cofactors for successful production of urease and carbonic anhydrase as both enzymes are metalloenzymes). The initial pH was adjusted to 8.0. Bioaugmented sets were inoculated with 0.5% inoculum (OD_600 nm_ = 1.0) of *Sporosarcina pasteurii ATCC 11859 (UB)* for ureolytic set and *Bacillus cereus C1 (CB)* for carbonic anhydrase set. Controls consisted of autoclaved sand without microbial supplementation to check the abiotic controls. Twelve AGW based enrichment media were prepared as follows:

**Table 2:** Details of enrichment medias in AGW used for current study.

**No**.	**Pathway**	**Media detail**	**Label**
**UREOLYTIC**			
1	Stimulation–Low carbon	YE (1 g/L) + Urea (100 mM) + NiCl_2_ (10 μM)	*BSTU-Low C*
2	Augmentation–Low carbon	YE (1 g/L) + Urea (100 mM) + NiCl_2_ (10 μM) + UB	*BAGU-Low C*
	Control- Low carbon		*CNT-Low C*
3	Stimulation–High carbon	YE (10 g/L) + Urea (100 mM) + NiCl_2_ (10 μM)	*BSTU-High C*
4	Augmentation–High carbon	YE (10 g/L) + Urea (100 mM) + NiCl_2_ (10 μM) + UB	*BAGU-High C*
	Control- High carbon		*CNT-High C*
**CARBONIC ANHYDRASE**			
1	Stimulation–Low carbon	YE (1 g/L) + NaHCO_3_ (100 mM) + ZnSO_4_ (1 μM)	*BSTC-Low C*
2	Augmentation–Low carbon	YE (1 g/L) + NaHCO_3_(100 mM) + ZnSO_4_ (1 μM) +CB	*BAGC-Low C*
	Control- Low carbon		*CNT-Low C*
3	Stimulation–High carbon	YE (10 g/L) + NaHCO_3_ (100 mM) + ZnSO_4_ (1 μM)	*BSTC-High C*
4	Augmentation–High carbon	YE (10 g/L) + NaHCO_3_ (100 mM) + ZnSO_4_ (1 μM) + CB	*BAGC-High C*
	Control- High carbon		*CNT-High C*

All the flasks were incubated at 37°C under shaking conditions at 50 rpm for 10 days. The controls contained autoclaved soils without any microbial supplementation.

### *In vitro* CaCO_3_ precipitation in flasks

In order to evaluate the effect of the microbial enrichments on efficacy of calcification, inoculum (1%) from all the enriched cultures from Section Culturing Conditions for Stimulation and Augmentation in Flasks was transferred to calcifying media (individual media in Table 2 supplemented with additional 50 mM CaCl_2_). Control samples were prepared without microbial inoculum. All the flasks were then incubated at 37°C in orbital shaker at 100 rpm for 10 days and monitored for pH, growth, enzyme production, extrapolymeric substance production, calcium precipitation and analysis of precipitated crystals.

### Chemical analysis and analytical methods for calcification in flasks

Changes in pH and growth were monitored. Growth was assessed by changes in optical density at 540 nm.

For the estimation of urea hydrolysis, enzyme production, extra polymeric substances (EPS) and soluble calcium content, culture filtrates were harvested, centrifuged (at 8,000 rpm for 15 mins) and the supernatant was analyzed. The hydrolysis of urea was estimated by the colorimetric urea analysis method as per Knorst et al. (1997). Briefly, 2 ml of the supernatant was mixed with 0.5 ml of 4% w/v p-Dimethyl benzaldehyde and 4% v/v sulphuric acid in pure ethanol. The mixture was incubated at room temperature for ten min and absorbance was taken at 422 nm against standard urea.

The urease activity was determined by measuring the amount of ammonia released from urea according to phenol-hypochlorite assay method (Dhami et al., 2013c). One unit of urease is defined as the amount of enzyme hydrolysing 1 μmol of urea per minute.

The carbonic anhydrase assay was performed as in Smith and Ferry (1999) with the modification prescribed by Yadav et al. (2010). One unit of carbonic anhydrase activity is defined as the amount of enzyme required to form 1 μmol of p-nitrophenol per minute.

The estimation of EPS was conducted as described by Bains et al. (2015) with minor modification. Around 20 ml culture from each set was centrifuged at 10,000 rpm at 4°C for 25 mins. The supernatant was collected and stored at −20°C for 1 h. EPS was precipitated by addition of three volumes of chilled absolute ethanol. The mixture was held at 4°C overnight and centrifuged at 10,000 rpm at 4°C for 15 min. The resulting pellet was dried at room temperature for 6 h followed by drying at 100°C until constant weight was obtained.

For carbonate precipitation efficacy, 10 ml supernatants were centrifuged at 4°C, filtered through 0.45 μm filter paper and analyzed for soluble Ca^2+^ concentration in the supernatant as well as insoluble CaCO_3_ in the precipitate by EDTA titration method as described in Stocks-Fischer et al. (1999). This was followed by quantification of calcium carbonate precipitates in each treatment at the end of the experiment. The contents of the flasks were filtered through 0.45 μm Whatman filter paper; washed with phosphate buffer saline solution and the filtrates were dried at 37°C for 12 h. The dry filtrates were weighed to measure the amount of crystals precipitated. These crystals were prepared for morphological and chemical characterization.

### DNA extraction

For DNA extraction, bacterial cells from all enrichments in Section Microorganisms Used in the Study were harvested by high speed centrifugation (5,600 rpm, 15 min) at 4°C and the pellet was used for DNA extraction using DNA extraction kit (PowerSoil™ DNA extraction kit, MO BIO Laboratories Inc.) following manufacturer's instructions. At the elution step, a small amount of DES (DNase/Pyrogen-Free Water) was used to recover DNA. The concentration of recovered genomic DNA was quantified using a Nanodrop 8,000 Spectrophotometer (Thermo Scientific, Wilmington, DE). Samples were diluted to a final concentration of 10 ng μL^−1^ to ensure sample standardization.

### 16S rRNA gene sequencing and bacterial population analysis

Microbial community analysis was done by high-throughput sequencing (MiSeq System—Illumina) using the primers, 341F (5′-CCTAYGGGRBGCASCAG-3′) and 806R (5′-GGACTACNNGGGTATCTAAT-3′) targeting V3-V4 variable region of the 16S rRNA gene. PCR amplification and sequencing was done at Australian Genome Research Facility (Brisbane, Australia). Briefly, PCR amplicons were generated using the primers and PCR conditions, as follows: 341F (CCTAYGGGRBGCASCAG) at 94°C and 30 s and at 50°C for 60 s and also 806R (GGACTACNNGGGTATCTAAT) for 29 cycles at 72°C and 60 s. AmpliTaq Gold 360 mastermix (Life Technologies, Australia) was used for the primary PCR. The secondary PCR to index the amplicons was performed with TaKaRa Taq DNA Polymerase (Clontech). The resulting amplicons were measured by fluorometry (Invitrogen Picogreen) and normalized. An equimolar pool was then created and quantified by qPCR (KAPA) followed by sequencing on the Illumina MiSeq with 2 × 300 bp Paired End Chemistry.

### Sequence analysis

Paired-ends reads were assembled by aligning the forward and reverse reads using PEAR (version 0.9.5) (Zhang et al., 2014). Primers were trimmed using Seqtk (version 1.0) (https://github.com/lh3/seqtk). Trimmed sequences were processed using Quantitative Insights into Microbial Ecology (QIIME 1.8) (Caporaso et al., 2010) USEARCH (version 7.1.1090) and UPARSE (Edgar et al., 2011) software. To obtain the number of reads in each OTU, reads were mapped back to OTUs with a minimum identity of 97%. Using Qiime taxonomy was assigned using SILVA database (version silva_119) (Quast et al., 2013). The obtained sequences were submitted to National Centre for Biotechnology Information (NCBI) (accession number SAMN06712353-12361) and further information is given in the Supplementary Material.

### Mineralogical and textural analysis

The micro textural features of the crystals precipitated by different biomineralizing conditions were observed by Scanning Electron Microscopy (SEM) (ZEISS EVO 50) equipped with Energy dispersive X ray spectrum (EDS). For the SEM analysis, the samples were fixed overnight in 2.5% glutaraldehyde in 0.1 M sodium phosphate buffer at 4°C, rinsed in 0.2 M phosphate buffer saline solution (pH 7.4) for 1 h and dehydrated in a series of graded ethyl alcohol. The SEM observation was done under the following analytical condition: EHT = 20.00 kv, WD = 10–11 mm. Elemental analysis was done with energy dispersive X-ray analyser (Bruker AXS, Quan Tax 200) to reveal the presence of individual elements in the samples. X-ray diffraction spectra (XRD) were obtained using an X' Pert PRO diffractometer with a Cu anode (40 kV and 30 mA) and scanning from 3 to 60° 2θ. XRD demonstrates the crystalline nature as well as phase composition (calcite, aragonite, vaterite, etc.). The components of the sample were identified by comparing them with standards established by the International Centre for Diffraction Data.

### Efficacy of biostimulation and bioaugmentation in sand columns

In order to determine the comparative efficacy of ureolytic and carbonic anhydrase pathways for biocementation applications in sands via stimulation and augmentation, the enriched consortias (OD = 1) grown in flasks were mixed into sterile autoclaved sand and casted in plastic syringes of diameter 25 mm and height 50 mm. Three sets of columns were designed as: Augmented, Stimulated and Control as per the methodology of Dhami et al. (2012). Briefly, 50 g sand was taken for each column and mixed with one pore volume of enriched consortia and casted into syringe column. In case of abiotic controls, the sand (autoclaved and sterile) was mixed with respective nutrient media for UA and CA set without microbial supplementation. As bacterial biomass and biofilms can also contribute to sand aggregation, another set of controls was made wherein the columns were fed without any supplementation of Calcium to avoid the production of carbonates. The bottom side of all the columns was blocked with whatman filter paper to avoid any loss of microbes. All the bacterial columns were next day flushed with 50 mM CaCl_2_ as a fixation fluid for bacterial attachment to the sand grains (Bernardi et al., 2014). After 6 h, all the columns were fed with one pore volume of their specific cementation media as 0.1 M Urea and 0.1 M CaCl_2_ for ureolytic sets while 0.1 M NaHCO_3_ and 0.1 M CaCl_2_ for CA sets. The cementation media was supplemented twice a day in all the sets for 10 days. At the end of the experiment, all the samples were dried at 50°C overnight, syringes were cut open with hot knife and the sand columns were taken out to visibly inspect the consolidation.

All the experiments were conducted in triplicates as biological replicates. The data were analyzed by Analysis of Variance (ANOVA) and the means were compared with Tukey's test. All the analyses were performed using Graph Pad Prism® software version 6.0.

## Results

### Effect of enrichment on pH, growth, enzyme activity, and EPS production in UA set

**Figure 1:**
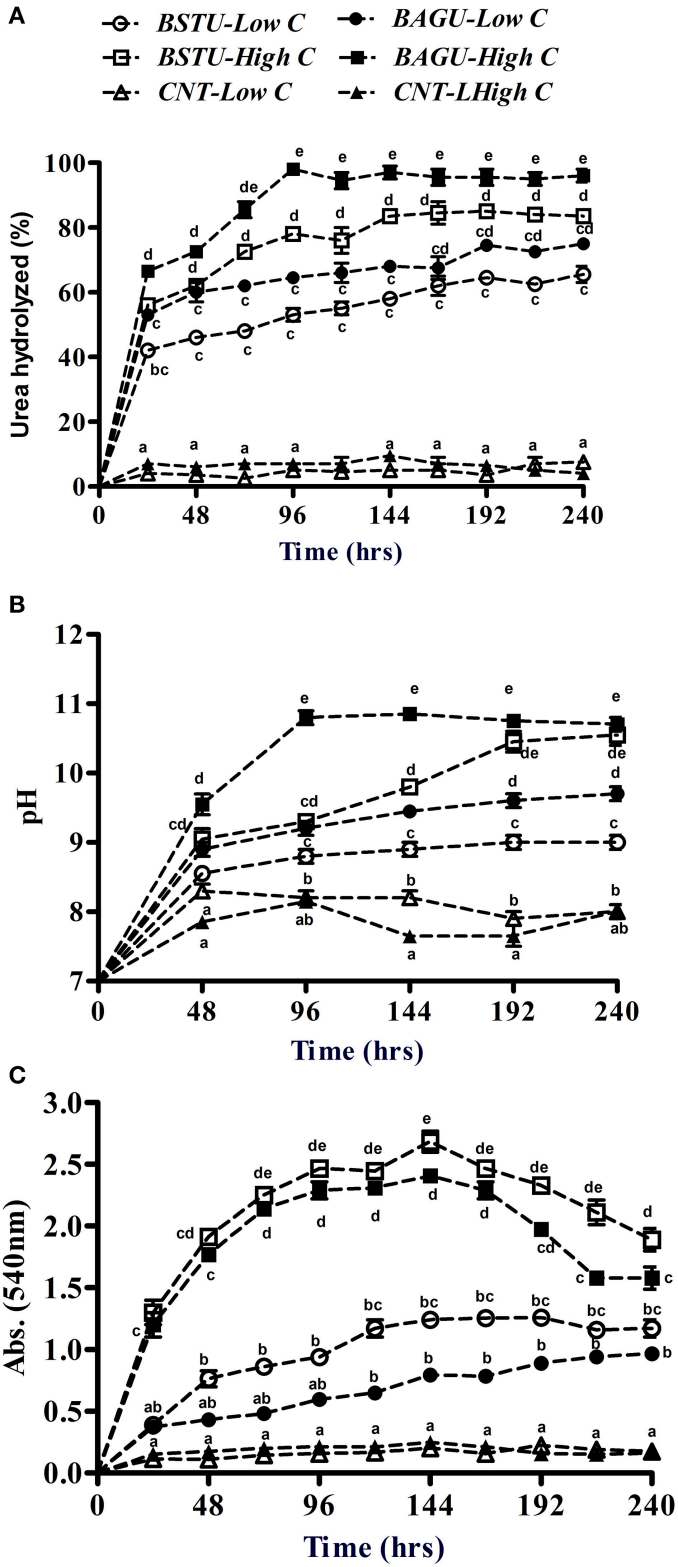
The effect of nutrient status of Biostimulated and Bioaugmented ureolytic enrichment cultures on the percentage of urea hydrolyzed **(A)**, pH evolution **(B)**, and optical density changes **(C)**. Values are mean ± SD (*n* = 3). The difference between means sharing a common letter is not statistically significant at *P* < 0.05.

Significant differences were recorded in the outcomes following enrichments under different conditions (Figure 1A). Following ureolytic enrichment, the urea hydrolysis was found to be highest in high nutrient augmented set (BAGU-High C) followed by high nutrient stimulated set (BSTU-High C), low nutrient augmented set (BAGU-Low C), and low nutrient stimulated set (BSTU-Low C). *S pasteurii UB* has been earlier reported to utilize urea highly efficiently and has the potential to grow in the presence of only urea (Bernardi et al., 2014; Gat et al., 2016) while most other communities require small dosage of organic carbon. Urea hydrolysis peaked at 96 h in high nutrient augmented set while in other cases it was steadily increasing till 192 h. There was an initial lag in urea hydrolysis under low nutrient conditions but effective urea hydrolysis was seen at later intervals. Efficient urea hydrolysis in the case of the stimulated sets indicates successful enrichments of the native ureolytic cultures. No changes were observed in any of the control sets.

All biotic ureolytic treatments resulted in an increase in pH ranging from 8.7 to 10.8 with negligible changes seen in the controls (Figure 1B). The pH changes reflected urea hydrolysis patterns with the highest pH change in high nutrient augmented and stimulated sets. Previous studies also indicated a positive correlation between urea hydrolysis and pH which is an indicator of potential MICP (Dhami et al., 2016b).

Growth, as measured by OD_540_, was greatest in the high nutrient sets (stimulated followed by augmented) compared to low nutrient sets which displayed poor growth (Figure 1C). Supplementation with a rich organic carbon source such as yeast extract significantly promotes the growth of microorganisms as it contains several growth factors including amino acids and peptides (Overmann, 2013). Contrary to the previous results of urea hydrolysis and pH, stimulated sets demonstrated slightly higher growth than augmented sets under both nutrient rich and poor conditions even though they had lower urea hydrolysis and pH earlier (Figures 1A,B).

**Figure 2:**
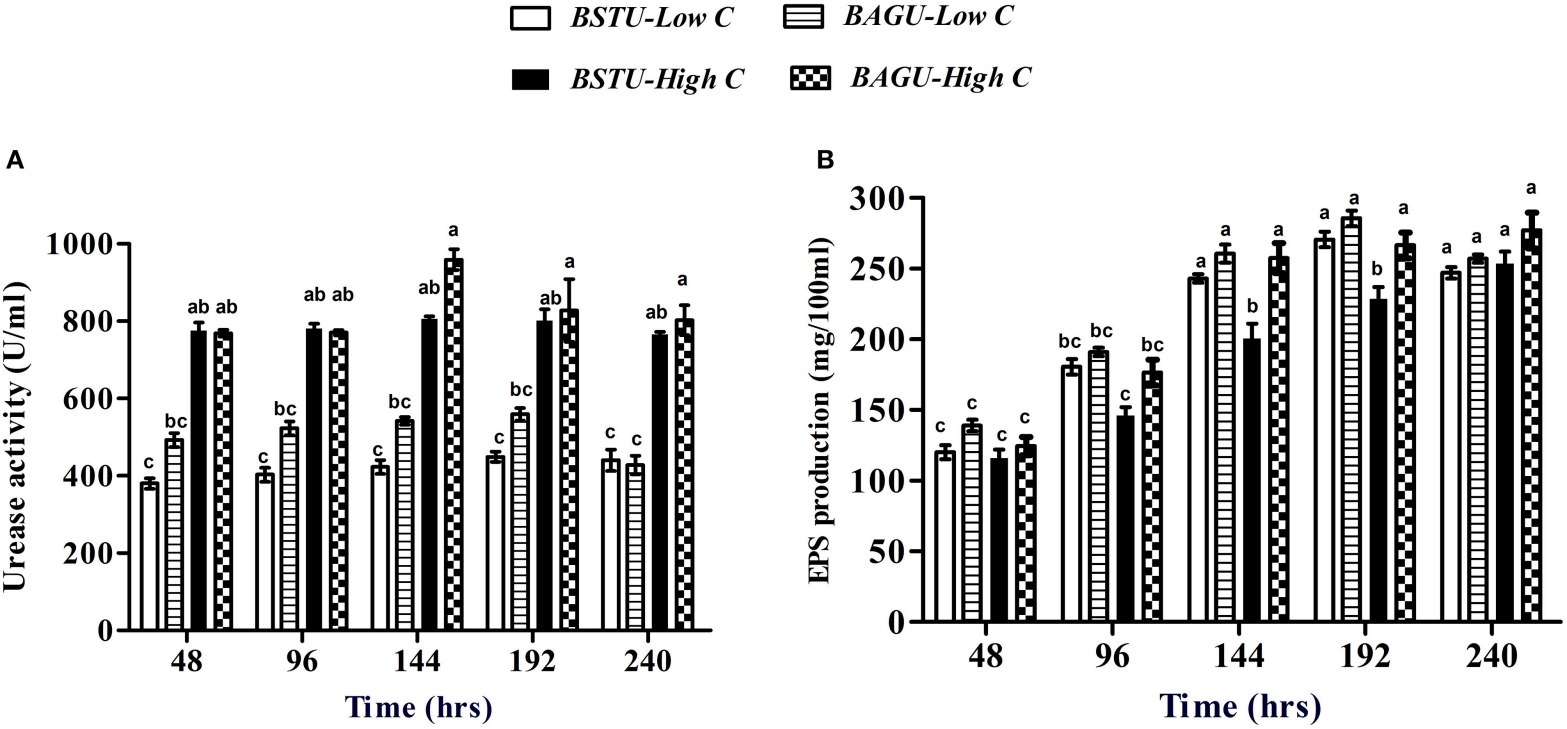
**(A)** Urease activity and **(B)** EPS production in different ureolytic enrichment media. Values are mean ± SD (*n* = 3). The difference between means sharing a common letter is not statistically significant at *P* < 0.05.

In case of urease sets the extracellular enzyme activity increased with an increase in the incubation time in all bacterial sets over a period of 144 h (Figure 2A). High nutrient augmented set BAGU-High C and stimulated set BSTU-High C showed the highest urease activities at 959.6 and 853.5 U/ml, respectively. The increase of urease with time is indicative of increase in biomass growth (Figure 1C) and release of the enzyme (Figure 2A). Higher activity in case of augmented sets compared to stimulated sets in low as well as high nutrient sets (after 144 h in high C sets) indicates that exogenous calcifying microbes as *S. pasteurii* may be competing initially but later acts synergistically with indigenous communities. In case of poor carbon environments, the urease production was significantly lower than high carbon nutrients. The low urease activity in case of low carbon sets of stimulation and augmentation could be due to lower biomass and hence little enzyme production in comparison to nutrient rich media.

The Extracellular polymeric substances (EPS) trend was quite variable in all the treatments (Figure 2B). The amount of EPS varied significantly from 92 mg/100 ml to 290 mg/100 ml in different sets. Low nutrient sets seemed to have higher EPS production compared to high nutrient sets in case of both stimulation and augmentation. It was recorded that the augmented sets displayed higher EPS compared to stimulated sets in both high and low nutrient conditions.

### Effect of enrichment on pH, growth, enzyme activity, and EPS production in CA set

**Figure 3:**
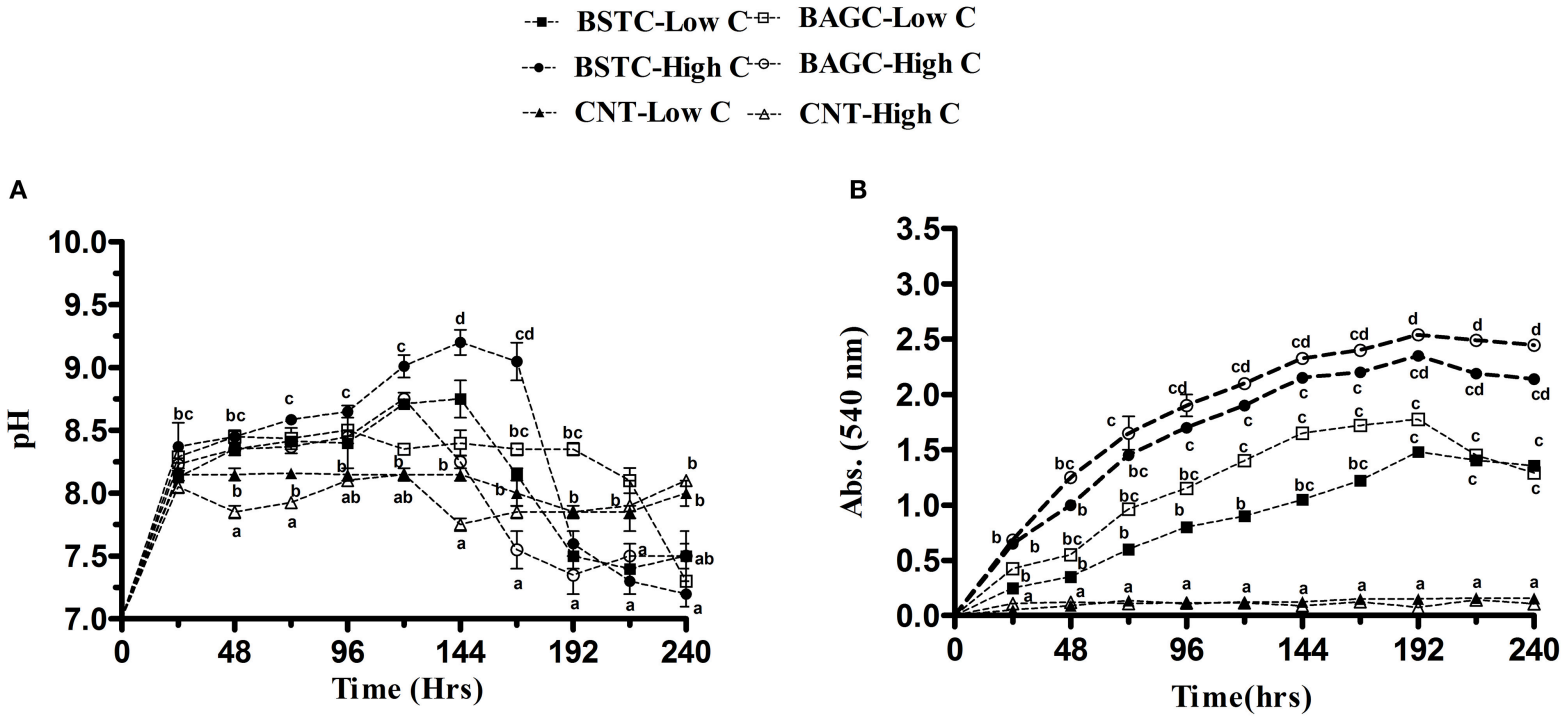
Evolution of **(A)** pH and **(B)** OD over time in different CA enrichment media with controls. Values are mean ± SD (*n* = 3). The difference between means sharing a common letter is not statistically significant at *P* < 0.05.

In case of carbonic anhydrase enriched sets, again varying outcomes were recorded (Figure 3A). pH was quite stable in sterile controls but varied noticeably in microbial sets. Compared to ureolytic sets, the pH was slightly lower. In case of high nutrients, the pH varied from 7.7 to 9.2 while in case of low nutrient sets, it varied from 7.4 to 8.7. In case of stimulated and augmented sets with high nutrients, pH was seen to increase in initial intervals but with time there was some decline. The pH variations in case of low nutrient sets were lower compared to high nutrient sets.

The optical density in case of microbial sets for carbonic anhydrase enrichment was noticed to demonstrate significant variations as it varied from 0.3 to 2.5 while there was no growth in the control sets (Figure 3B). Highest OD was noticed in case of high nutrient BAGC-High C set followed by BSTC-High C, BAGC-Low C, and BSTC-Low C. Compared to ureolytic enrichment, the growth in this case was slightly lower during initial hours in all sets though in later intervals, significant growth was seen especially in augmented sets. Smith and Ferry (2000) reported that CA enzyme is prevalent in different bacterial communities but till date very few studies have been done to evaluate its prevalence in bacteria from natural environments.

**Figure 4:**
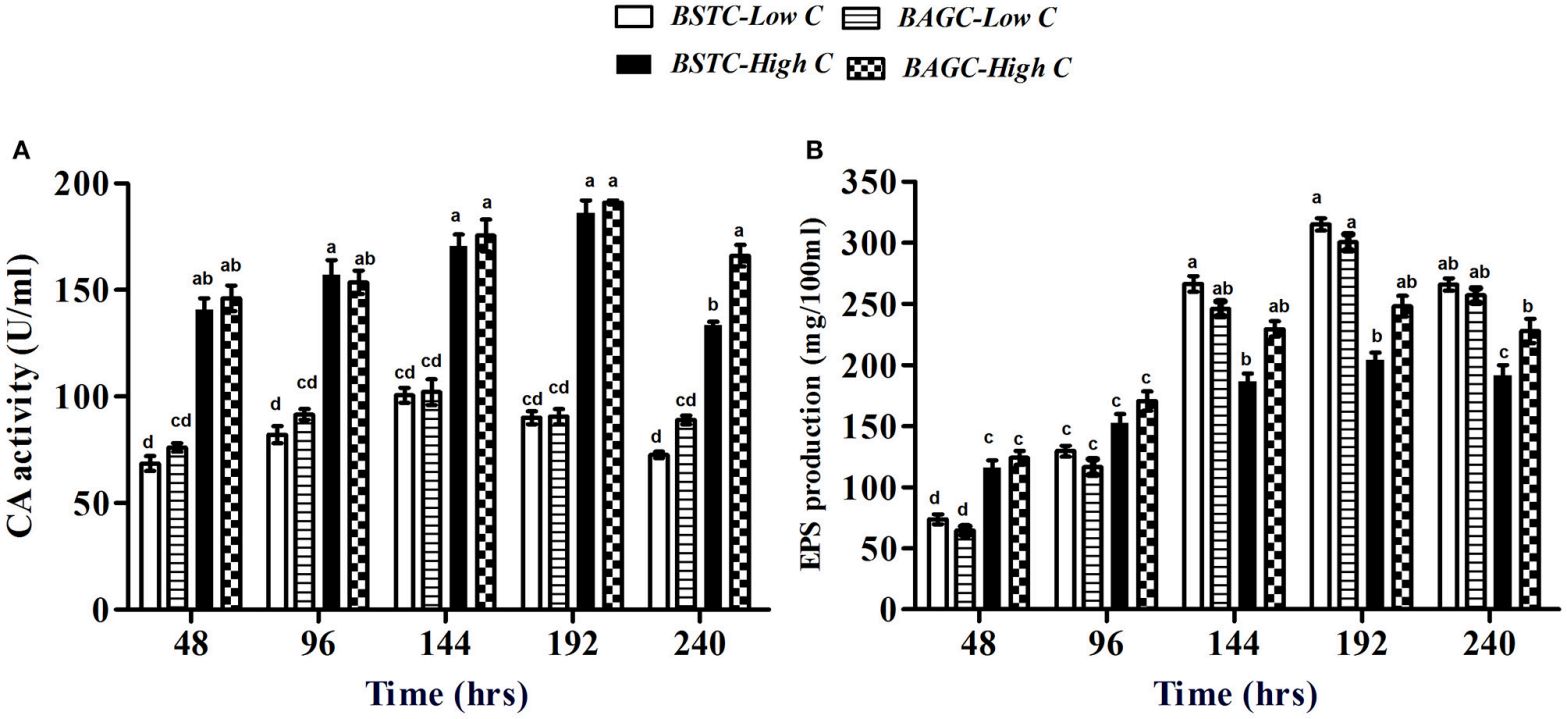
**(A)** Carbonic anhydrase activity and **(B)** EPS production in different CA enrichment media. Values are mean ± SD (*n* = 3). The difference between means sharing a common letter is not statistically significant at *P* < 0.05.

The production of carbonic anhydrase enzyme in different enrichment sets is presented in Figure 4A. Increase in enzyme production commenced in all the microbial enriched sets with significant variations amongst different treatments. Higher CA production was recorded in high nutrient sets compared to low nutrient sets. Highest enzyme production was noticed in BAGC-High C set followed by BSTC-High C, BAGC-Low C, and BSTC-Low C again demonstrating higher activity in augmented sets compared to stimulated sets. The trend followed a similar pattern as that of urease production (Figure 3A).

The extrapolymeric substances production in case of CA sets is presented in Figure 4B. The amount of EPS in this case varied from 76 mg/100 ml to 320 mg/100 ml over the period of study. The trend in descending EPS amounts was BSTC-Low C, BAGC-Low C, BAGC-High C, and BSTC-High C. Higher EPS production was noticed in case of low nutrient environments compared to higher ones similar to ureolytic sets (Figure 3B). In this case, slightly higher EPS was seen in case of stimulated set under low nutrient conditions while augmented sets produced higher EPS under high nutrient conditions.

### Effect of enrichment on calcium precipitation

**Figure 5:**
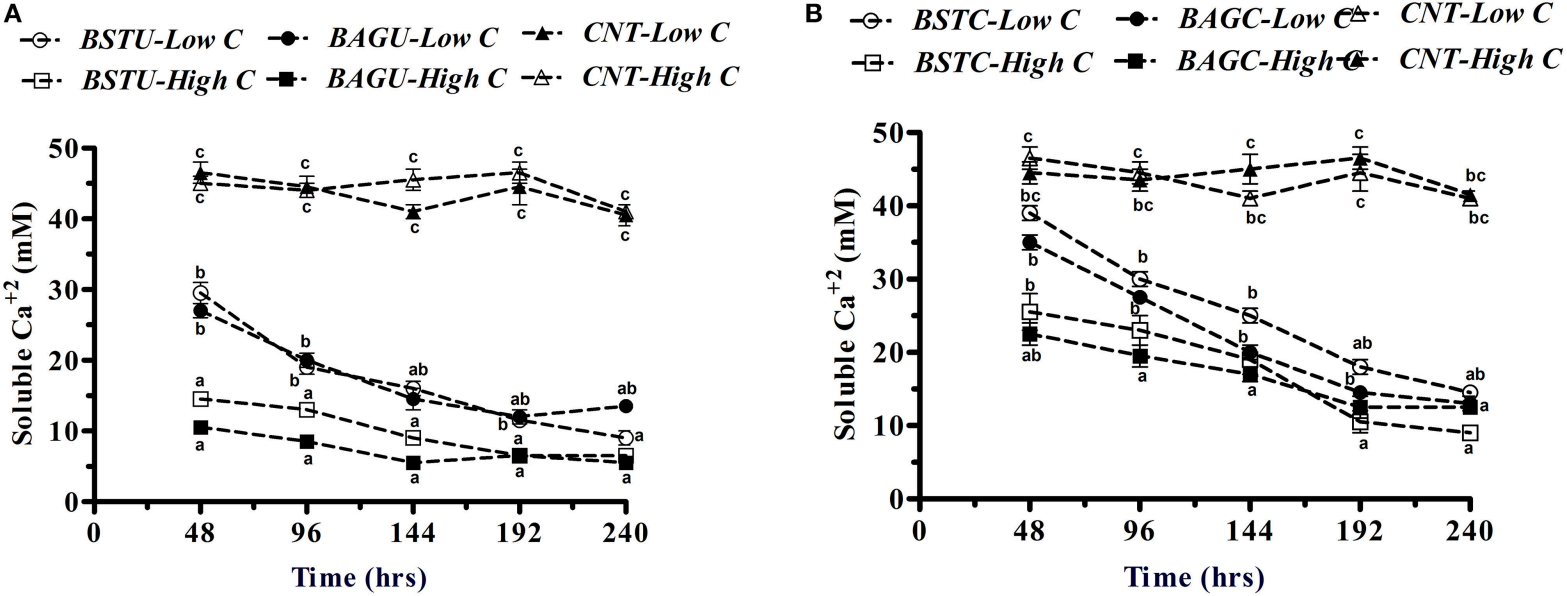
Soluble calcium content among different **(A)** ureolytic and **(B)** carbonic anhydrase enrichment media. Values are mean ± SD (*n* = 3). The difference between means sharing a common letter is not statistically significant at *P* < 0.05.

In the case of Ureolytic sets, a declining trend in soluble calcium content was noticed in all bacterial enrichment cultures with time (Figure 5A). The trend of calcium removal seemed to follow a similar route as urea hydrolysis although low nutrient sets displayed significant calcium consumption too despite of lower enzyme production and metabolic activity. The descending trend for Calcium was as BAGU-High C, BSTU-High C, BSTU-Low C, and BAGU-Low C. In case of BAGU-High C set, more than 80% calcium removal (carbonate precipitation) occurred in first 2 days. Slightly lower amounts were seen in case of BSTU-High C sets (upto 72%). Initially the calcium precipitation in both sets with lower nutrients was slow but with time, significant decline in soluble calcium content was noticed. Contrary to urea hydrolysis, high Calcium precipitation in case of lower nutrients might be attributed to significant EPS formation in low nutrient conditions as seen in Figure 3B. Not much variation in Calcium content was recorded in control sets hinting little abiotic precipitation.

In case of Carbonic anhydrase sets (Figure 5B), the soluble Calcium content removal revealed that considerable removal occurred in all bacterial sets with time and insignificant changes occurred in controls here also. Compared to ureolytic sets, the trend of soluble Calcium removal and insoluble Calcium carbonate precipitation was comparatively slower in case of CA sets. The soluble Calcium content decreased faster in high nutrient sets BAGC-High C and BSTC-High C compared to lower sets during initial intervals. Around 60% Calcium removal and precipitation was seen in case of BAGC-High C set while around 50% precipitation occurred in BSTC-High C sets in initial 48 h. In case of BSTC-Low C and BAGC-Low C sets, the carbonate precipitation was slower initially upto 96 h (around 20–36%) but later on it was found to increase reaching upto 70% in 192 h in case of BAGC-High C and 58% in case of BSTC-High C. This also might be in relation to the carbonate precipitation through EPS pathway under poor nutrient environments.

All these experiments confirmed that both active (through metabolically active routes under high nutrients) and passive (through EPS production under low nutrients) routes can play substantial role in promoting Calcium carbonate precipitation. Though bioaugmentation was seen to be more effective than stimulation in both ureolytic and carbonic anhydrase enrichment routes in terms of extracellular enzyme production and activity, however, the stimulation of native communities seems promising too. In the next part we investigated different microbial communities which were actively metabolizing under experimental enrichments.

### Analysis of microbial diversity

**Figure 6:**
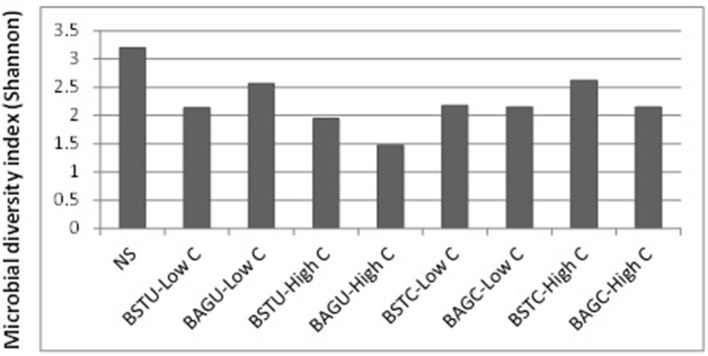
Shannon index diversity of different enrichment treatments compared to native sand.

The diversity of microbial communities under different enrichments was analyzed to investigate the predominance of active communities and examine the potential of augmented cultures to survive along with the native microbes. The sequences were submitted to NCBI and were grouped into Operational taxonomic units and the classification was against Greengene data base which provides the community resolution upto genus/ species level. The information on OTUs and number of reads per sample is available in the Supplementary Material. Figure 6 represents the Shannon index diversity for each treatment as well as native sand at genus level. It was recorded that the microbial diversity decreased in all the enrichments compared to native sand. Lowest amount of diversity was seen in case of augmented ureolytic set under high nutrient conditions which indicates dominance of a few communities only.

**Figure 7:**
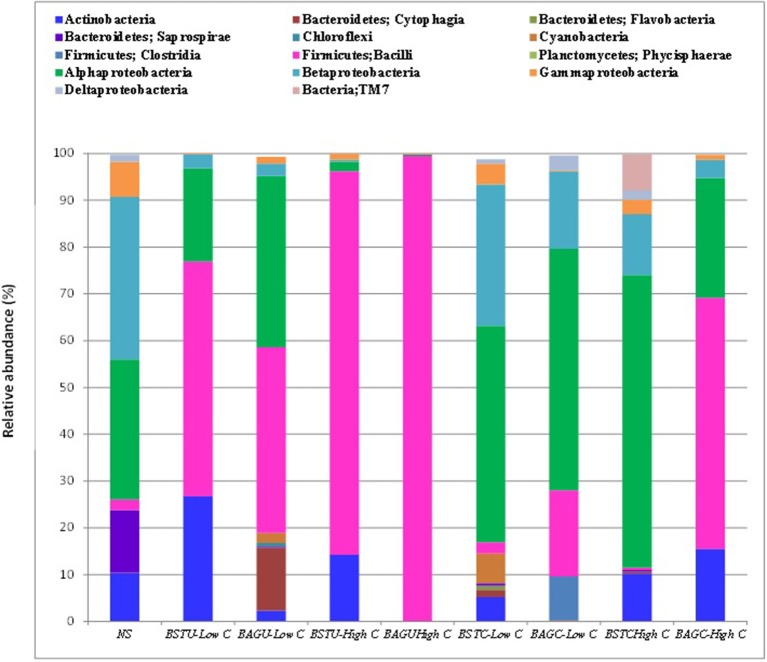
Bacterial population composition based on 16S rRNA gene sequencing presented as a fraction of each bacterial class from total population.

**Figure 8:**
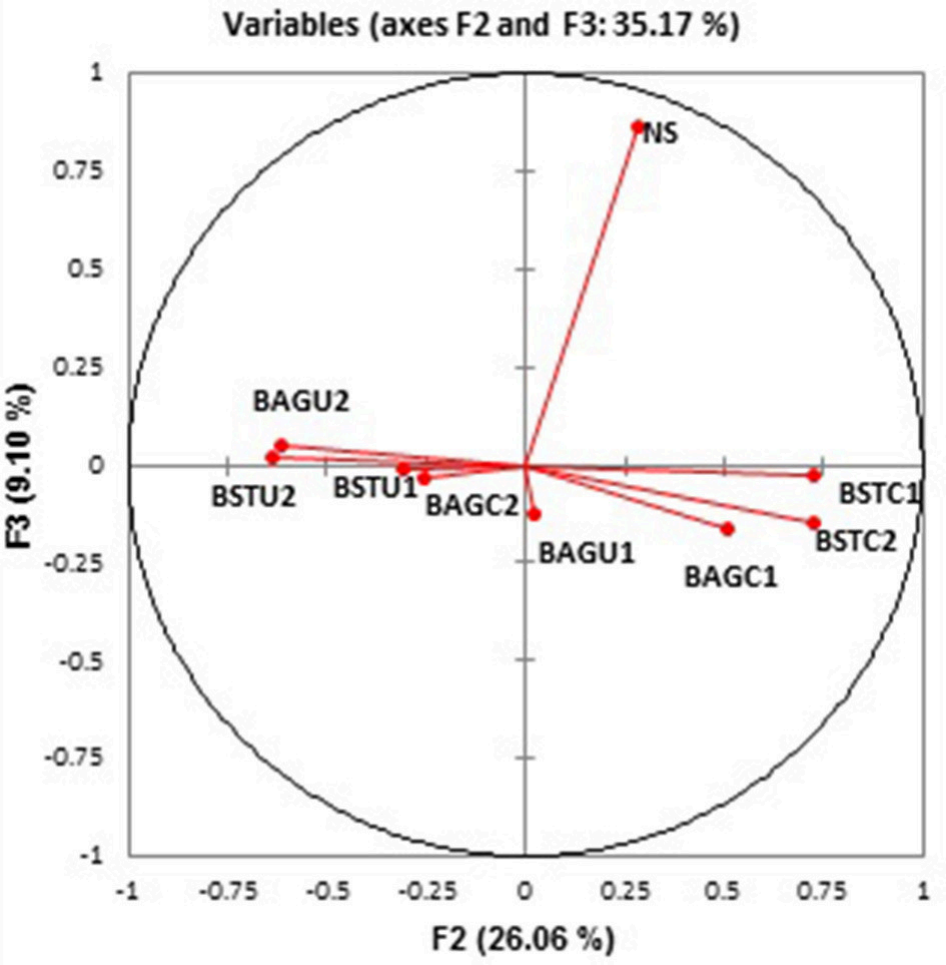
PCoA analysis based upon weighted Unifrac distances for different enrichment treatments.

Figure 7 represents the relative abundance of microbial communities at Class level and Table 3 represents relative abundance of the dominant microbial communities in all treatments at genus level compared to the native sand. Tremendous variations in the bacterial compositions under different enrichments and routes were recorded. The effect of native microbial communities and competitions with augmented bacterial cultures *Sporosarcina pasteurii UB* and *Bacillus cereus CB* showed evident outcomes. The influence of nutrient conditions seemed to play significant role not only in biomass determination (Figures 1C, 2B) but also bacterial diversity. Distinct clustering was also observed via PCoA analysis as per the enrichment media composition and nutrient source despite similarities (Figure 8). Interestingly, the native sand clustered separately from all treatments indicating the dissimilarity and alterations in microbial communities upon enrichments under different conditions. More diversity variations were recorded in case of ureolytic sets compared to carbonic anhydrase sets in this study.

**Table 3:** Heat map of dominant microbial communities at genus level with their relative abundance within the community in all treatments and the native sand (in percentage).

**Taxon**	**NS**	**BSTU-Low C**	**BAGU-Low C**	**BSTU-High C**	**BAGU-High C**	**BSTC-Low C**	**BAGC-Low C**	**BSTC-High C**	**BAGC-High C**
**Leucobacter (Actinobacteria)**	0.18	25.97	1.81	13.23	0.03	4.98	0.1	0.76	10.37
**Pontibacter (Bacteroidetes)**	0.02	0	13.46	0	0	1.52	0.03	0.42	0
**Flavihumibacter (Bacteroidetes)**	10.7	0.02	0	0.01	0.01	0	0	0.07	0
**Unclassified Bacillus (Firmicutes)**	0.97	27.18	15.7	19.5	23.4	0.12	0.56	0.07	31.54
**Planococcus (Firmicutes)**	0.05	1.59	0.26	37.36	25.3	0.01	0.21	0.03	3.32
**Lysinibacillus (Firmicutes)**	0.63	1.15	0.1	0.31	0	0.05	10.34	0	3.56
**Sporosarcina (Firmicutes)**	0.06	1.71	21.67	11.53	43.77	0.05	6.1	0.13	0.55
**Brevundimonas (Proteobacteria)**	0.66	0.02	0.07	0.02	0.02	2.48	30.42	1.14	22.01
**Ochrobactrum (Proteobacteria)**	0.72	19.66	8.19	0.1	0.04	8.66	4.65	0.96	1.55
**Methylobacterium (Proteobacteria)**	0.27	0.02	0.06	0.01	0.04	0.06	0	33.55	0.03
**Phyllobacter (Proteobacteria)**	4.75	0.09	9.2	0.03	0	3.27	14.72	3.91	1.57
**Sphingomonas (Proteobacteria)**	4.61	0	7.66	0.02	0.03	16.77	0.02	1.71	0.02
**Alcaligenes (Proteobacteria)**	0.04	2.57	0.45	0.32	0.04	27.68	16.48	11.79	3.67
**Unclassified Comamonadace (Proteobacteria)**	31.41	0.02	0	0	0	0.03	0	0.02	0.02
**Providencia (Proteobacteria)**	0	0.01	1.05	0	0	0.03	0	0	0

*Abundance scale (Green, High; Yellow, Medium; Red, Low)*.

In case of native sand, the most abundant bacterial class was Betaproteobacteria (34.7%) followed by Alphaproteobacteria (30%), Bacteroidetes (13.4%), and Gammaproteobacteria (7.4%). The Bacilli Firmicutes comprised only around 2% of total populations. Ureolytic biostimulation led to significant alteration to the microbial population. The bacterial population was quickly overtaken by Firmicutes in most of the UA enrichments. At low nutrient conditions, stimulation led to increase of Firmicutes (Bacilli) upto 50% followed by Actinobacteria and Alphaproteobacteria. The most predominant Genus identified in this case was Leucobacter (Actinobacteria). In case of augmentation, Firmicutes were again dominating with a proportion of 39%. At genus level, *Sporosarcina pasteurii UB* was seen to cover a proportion of 21.6% followed by other *Bacillus* sp. in case of low nutrient conditions. At high nutrient levels, the growth of Firmicutes was promoted and it entirely overtook the bacterial population upto 81% indicating the success of stimulation in enriching ureolytic cultures. The predominant genus identified in this case was *Planococcus* and other *Bacillus* sp. This indicates that the growth and enrichment of native ureolytic communities amongst plethora of other microbes is quite promising. In case of augmented set, again Firmicutes successfully dominated to 99.3%. *S pasteurii UB* in this case dominated with a proportion of 43% followed by *Planococcus* which is another Gram positive *Bacillus* sp. Our study revealed that under high carbon conditions, enrichment of indigenous ureolytic microbes is highly successful. The effect of nutrient conditions also promoted the survival and dominancy of *S. pasteurii UB* confirming the successful survival and competence of this culture in presence of other native communities. Recent reports of Phillips et al. (2016) also confirmed the survival of augmented *Sporosarcina pasteurii* in deep soils where it successfully overtook native *Pseudomonas* sp. This study further confirms the promising potential of this culture for several bio-engineering applications involving augmentation.

In case of carbonic anhydrase sets also, nutrient conditions and enrichment technique displayed significant variations. Low nutrient conditions promoted the enrichment of Alphaproteobacteria class which reached 46% followed by Betaproteobacteria at 30% in case of BSTC-Low C set. The Firmicutes in this case were not enriched. The most predominant genus in this case was Alcaligenes and Sphingomonas. In case of augmented set BAGC-Low C, again Alphaproteobacteria were predominant at 51% but in this case, Firmicutes were also enriched at 18%. The dominant genus in this case was Proteobacteria *Brevundimonas* along with enrichment of *Bacillus*. Under high nutrient conditions of stimulation, Alphaproteobacteria were found to dominate at 62% with the dominate genus *Methylobacterium* at 33.5% while in case of augmented set, Firmicutes dominated at 53% with dominance of *Bacillus* at 31.5%. Though the augmented culture *Bacillus cereus CB* could not be identified upto species level but interestingly, dominance of Firmicutes might be indicative of survival of the supplemented culture along with other Bacillus species under high nutrient conditions indicating the potential and efficacy of this culture. Though compared to augmented ureolytic culture prevalence, the survival of CA isolate in augmentation is quite low, but further investigations on improving the survival of augmented cultures through varying media components and immobilization techniques or using other gram negative CA producing isolates from Phylum Proteobacteria can be explored. Smith and Ferry (2000) recently reported that CA producing microbial cultures are prevalent in phylum Proteobacteria while Srivastava et al. (2015) isolated high CA producing gram negative isolate *Serratia* sp. from marble rock soils.

### Characterization of precipitated crystals

**Figure 9:**
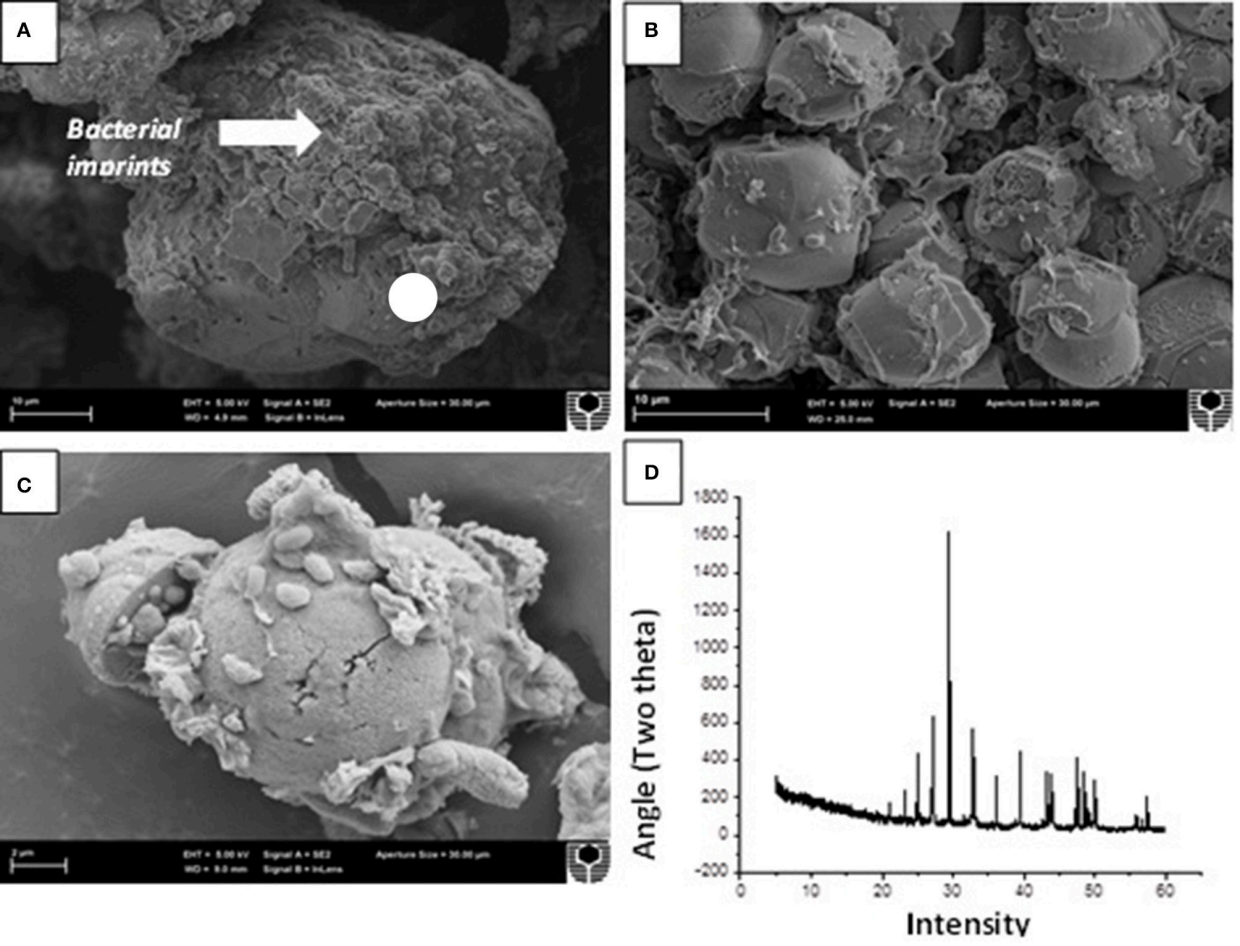
Scanning electron microscopy of crystals precipitated in **(A)** BSTU-High C **(B)** BSTU-Low C **(C)** EPS formation in low nutrient set BAGU-Low C, and **(D)** X ray-diffraction analysis of carbonate crystals precipitated in BAGU-High C depicting presence of calcite and vaterite.

The crystals precipitated under different enrichment conditions followed by supplementation of soluble calcium chloride were then analyzed through microscopy and mineralogical analysis (Figure 9). SEM analysis clearly depicted the formation of varying sized crystals in all the microbial enrichments. In general, the crystals were mostly smooth, rhombohedral and spherical with sizes varying from 30 to 150 μm under high nutrient conditions while in case of low nutrient conditions; small round crystals were predominant with sizes between 10 and 50 μm irrespective of augmentation and stimulation (Figures 9A,B). Some slimy EPS formation was noticed in samples with low nutrient conditions under both ureolytic and CA routes (Figure 9C). The presence of crystals associated with bacterial cells and EPS confirms their role as nucleation sites during mineralization process and has been demonstrated by several previous studies (Bergdale et al., 2012; Bains et al., 2015). X ray diffraction analysis of all the crystals was also carried out and various polymorphs were seen in different enrichment sets (Figure 9D). Biostimulation as well as augmentation in case of UA route led to predominance of calcite crystals under high nutrient conditions while in case of CA enriched sets, mix of different polymorphs as calcite, vaterite and aragonite was seen.

### Microbially cemented sand plugging

**Figure 10:**
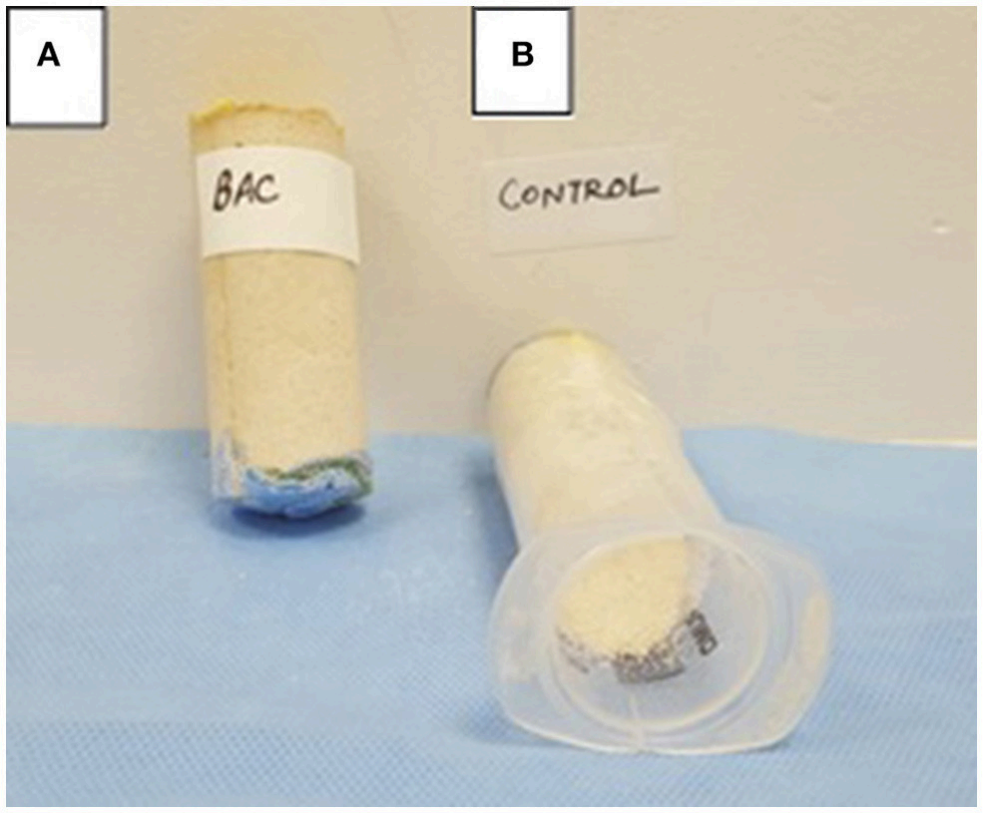
**(A)** Bacterially cemented sand column bioaugmented with *S. pasteurii* and **(B)** Abiotic control sand column.

In order to further check the efficacy of MICP via UA and CA routes under stimulation and augmentation conditions inside sand columns, the extent of consolidation was investigated visually after 10 days. It was noticed that all the Calcium supplemented and enriched sand columns via UA and CA consortias were highly consolidated while the abiotic and no Calcium treated controls collapsed immediately after opening the plastic column. Higher consolidation/compactness was noticed in case of ureolytic consortia enrichment compared to CA enrichment under both stimulation and augmentation conditions. In general, *S. pasteurii* augmented enrichment led to the highest consolidation (Figure 10) compared to other sets. The results of sand columns were supportive of the previous flask results wherein more effective Calcium precipitation was observed in case of ureolytic sets. Further studies need to be conducted for determining the effect of various treatments on mechanical and permeability properties of granular materials through geotechnical testing at large scales.

## Discussion

In the current study, we enriched microbial communities in calcareous soil via two different routes of carbonate mineralization: ureolytic and carbonic anhydrase for the first time. We compared the efficacy of biostimulation with respect to bioaugmentation wherein standard cultures were supplemented along with the native communities. The effect of nutrient conditions of high carbon and low carbon was explored as all these parameters affect the efficacy of MICP in biocement applications for geotechnical engineering. The effect of different enrichment conditions was checked on various parameters including biomass, enzyme activity, EPS production, calcium precipitation, microbial community dynamics, carbonate polymorph selection and biocement consolidation.

In case of ureolytic enrichment, highest urea hydrolysis and biomass was recorded under bioaugmentation conditions with high nutrient conditions. This might be because of the potential of supplemented strain *S. pasteurii UB* which is capable of effectively utilizing urea compared to other cultures. Significant urea hydrolysis and biomass was observed through biostimulation proving the potential and metabolic activity of native ureolytic communities. Urea is an organic nitrogenous compound present in several coastal environments which has been introduced by the excreta of terrestrial and aquatic animals (Gat et al., 2016). So, utilization of urea as energy source is quite predominant in those environments paving the way to significant occurrence of urea hydrolysing communities. This may not be possible in the case of low organic matter environments (Gat et al., 2016). Notable urease production was also measured under high nutrient conditions with both stimulation and augmentation. It has been found that extracellular urease is adsorbed onto clay minerals and organic matter in soils where its activity is stable for years (Nannipieri et al., 1983). Even under low nutrient conditions, some fraction of urea hydrolysis and urease production was noticed. This extracellular urease might be contributing to urea hydrolysis under low nutrient conditions. Though higher growth was seen in case of stimulation but urea hydrolysis was lower compared to augmentation hinting that this could be due to varying hydrolytic efficacy of different communities as not all hydrolysing communities are as effective as *S. pasteurii UB*.

In case of CA route also, successful enrichment of indigenous communities was achieved under high nutrient conditions via stimulation. Higher biomass and CA production was recorded in case of augmentation indicating that augmented cultures might be acting synergistically along with indigenous communities. This is the first study on enrichment of CA communities from soils utilizing bicarbonates with efficacy for carbonate precipitation. Present results demonstrated that enrichment of these communities through stimulation can be favored by use of bicarbonates along with supplementation of complex organic carbon source as yeast extract.

Significant EPS production was noted in case of low nutrient conditions via stimulation as well as augmentation under both enrichment conditions of UA and CA metabolism. Previous studies of Myszka and Czaczyk (2009) demonstrated higher EPS production by *Psedomonas aeruginosa* under starvation conditions which was also supported by González-García et al. (2015) and Evans (2013). The higher EPS may be indicative of microbial defense under stressed conditions promoting higher biofilm matrix. The formation of EPS has been found to play a significant role in Calcium carbonate precipitation in a number of previous studies (Bergdale et al., 2012; Ercole et al., 2012).

Efficient calcium carbonate precipitation was measured under UA as well as CA route not only under high nutrient conditions with higher extracellular enzyme production but also under low nutrient conditions. In general, augmented sets in UA and CA had higher Ca^2+^ precipitation compared to stimulated sets which indicates synergism between the indigenous communities and the augmented bacterial isolates. Significant precipitation of calcium carbonate in all stimulated sets indicates the promising potential of this route. Under low nutrient conditions, the carbonate precipitation seemed to follow the EPS route. Previous studies have also found that EPS plays significant role in Calcium carbonate precipitation and is quite common pathway for formation of several natural formations as stromatolites, mats, beach rocks etc. (Ercole et al., 2007). We also reported a direct correlation between EPS concentration and calcium carbonate precipitation by *B. megaterium SS3* in previous studies (Dhami et al., 2014b).

A number of previous studies have been conducted which successfully demonstrated the potential of ureolytic communities in carbonate precipitation but only a few studies have yet explored the calcium carbonate precipitation potential of CA producing bacterial cultures (Dhami et al., 2014b, 2016a; Srivastava et al., 2015). This study has demonstrated the capability of indigenous bacterial cultures to utilize bicarbonate ions during carbonate precipitation (Bharti et al., 2014; Dhami et al., 2014b; Kaur et al., 2016). Srivastava et al. (2015) reported that in shake flask culture conditions, carbon dioxide sequestration and sodium bicarbonate dissociation follow the following biochemical reactions:

$$\begin{array}{lll} {\text{H}}_{2}\text{O} & \to & {\text{H}}^{+}+{\text{OH}}^{-}\\ 2{\text{CO}}_{2}+{\text{OH}}^{-} & \to & {\text{HCO}}_{3}^{-}+{\text{CO}}_{2}\\ {\text{CaCl}}_{2} & \to & {\text{Ca}}^{2+}+2{\text{Cl}}^{-}\\ {\text{Ca}}^{2+}+\text{Bacterium} & \to & \text{Bacterium}-{\text{Ca}}^{2+}\\ {\text{NaHCO}}_{3}^{-} & \to & {\text{Na}}^{+}+{\text{HCO}}_{3}^{-}\\ {2\text{HCO}}_{3}^{-}+{\text{Bacterium}-\text{Ca}}^{2+} & \to & {\text{Bacterium}-\text{CaCO}}_{3}\\ + & {\text{CO}}_{2}+{\text{H}}_{2}\text{O} \end{array}$$

The outcome of this experiment highlighted the feasibility of enriching native CA communities under high nutrient conditions for effective microbial carbonate precipitation. In terms of the efficacy of CA route compared to ureolytic route, though this route is less effective but its substantial advantage is no ammonia production which restricts the use of ureolytic pathways under few circumstances.

Microbial community dynamics have received very poor attention in previous studies of biocementation applications in different soils. Previous studies of Kostka et al. (2011), Whitman et al. (2014) and Gat et al. (2016) reported that Proteobacteria are quite dominant in beach sands, coastal sands, marine and fresh waters. Even in this study we noticed the prevalence of this phylum in native sands but followed by enrichments, tremendous variations in microbial dynamics appeared. In case of UA enrichments of high C conditions, Firmicutes exhibited relative abundance. Large number of studies have demonstrated the potential of *Bacillus sp*. from Firmicutes in urea hydrolysis as Bacilli are common in alkaline environments and their increase under ureolytic environments is associated with their urea hydrolysing potential (Burbank et al., 2012; Dejong et al., 2013; Dhami et al., 2013c). Under stimulation conditions, native *Bacillus* sp. *Planococcus* was prevailing the most while under augmentation, *S. pasteurii UB* was seen to dominate. The robustness, high metabolic activity and stability of this strain has led to its emergence as a highly promising calcifying culture for biocementation applications and this study further confirmed the potential of this isolate to easily adapt, survive under competition and actively drive calcification in presence of the native communities. Though under low carbon conditions, the augmented isolate did not predominate but its presence indicated its efficacy. Similarly in case of CA route, Proteobacteria was seen to be the predominating phylum under most of the enrichments. Though very little has been reported on microbial diversities associated with CA route in soils, few studies demonstrated that microbial communities from Proteobacteria have widespread carbonic anhydrase production (Dobrinski et al., 2010; Ueda et al., 2012; Bharti et al., 2014). Only in case of high C conditions, the augmented culture *B. cereus CB* was seen to survive while in all other enrichments, bacterial isolates belonging to Proteobacteria were prevailing suggesting that further investigations should be made to explore other communities. Biostimulation as well as augmentation through ureolytic route was recorded to be comparatively more effective compared to carbonic anhydrase route but, as successful enrichment of CA communities associated with MICP along with their effective metabolism leading to significant calcification was achieved, this route seems to have immense potential.

The nutrient conditions seemed to play a very significant role in determining the survival and efficacy of augmented cultures along with their composition. This might be due to the fact that rich organic carbon media promotes not only growth but also the concentration of DIC which further impacts pH and hence microbial compositions selection (Rodriguez-Navarro et al., 2012). Synergism between augmented UA as well as CA cultures is also quite noticeable under nutrient rich environments as both the isolates were seen surviving along with the indigenous communities. Under low nutrient conditions, Firmicutes comprising the augmented cultures were not enriched significantly compared to their prevalence under high nutrients in UA set. This might be indicative of better acclimatization of the indigenous communities under those environments. Compared to augmented CA isolate *Bacillus cereus CB* isolate, UA isolate *Sporosarcina pasteurii UB* bears more chances of survival and existence under low nutrient conditions making it more robust and attractive for field applications.

Upon characterization of the carbonates formed under varying enrichment conditions, huge variations were noticed. This could be demonstrated on the basis of varying environmental conditions including DIC, growth medium composition, bacterial metabolic activity, saturation index which influence the type and properties of calcium carbonate polymorphs. Previous studies have also recorded that the fate of calcium carbonate polymorph is dependent upon a number of factors as composition of growth medium, type of substrate, temperature, pH, saturation index, [Ca^2+^]/[${\text{CO}}_{3}^{2-}$] ratio, bacterial species, and organic matter (Rodriguez-Navarro et al., 2012; Dhami et al., 2013c). Availability of nutrients seemed to play the most important role in determining the fate of carbonates. In this case also predominance of calcitic crystals was noticed under UA route of high C augmentation supporting the immense potential of this pathway for civil engineering applications. The outcome of these results established that biomineralization through UA and CA routes via stimulation as well as augmentation is an effective and eco-friendly route for synthesis of carbonates but availability of nutrients have tremendous impact on the potential of whole process as it influences the microbial growth and metabolic state which further affects pH and the saturation index affecting the formation of different carbonate polymorphs.

Finally, the biocementation effect of enrichments was investigated through sand column tests. In a preliminary investigation, it was recorded that successful consolidation of sand plugs was achieved through microbial routes with supplementation of Calcium rich cementing media but UA enriched pathways lead to higher carbonate precipitation. Amongst all, UA enriched sand columns supplemented with *S. pasteurii UB* illustrated the highest consolidation. This once again, supports the supremacy of this culture over other calcifying isolates as it has high potential to metabolize under only urea supplemented source. As visually noticeable sand plugging was recorded in augmented CA sets also, this route needs to be explored further for sustainable applications.

## Conclusion

This is the first study to investigate and compare microbial induced carbonate precipitation potential via stimulation and augmentation through UA and CA routes under low and high carbon conditions. It was demonstrated that biostimulation as well as augmentation through ureolytic route is comparatively more effective compared to carbonic anhydrase route but, as successful enrichment of CA communities associated with MICP from native populations was achieved, this route needs to be explored further. Firmicutes were seen to be the dominating communities under UA enrichments while Proteobacteria dominated under CA sets. Successful biostimulation of UA and CA communities can render augmentation unnecessary and simplify various field scale applications of MICP. Interestingly, augmentation was seen to be more effective under high nutrient conditions in both UA and CA route indicating synergistic role of augmented isolates along with indigenous communities. The role of nutrient availability also highlighted its influence on carbonate polymorph as large calcitic crystals were prevalent in case of high nutrients while smaller crystals were recorded more in low nutrient sets. The outcome of this work states that stimulation can be favored at sites with high organic carbon content while augmentation with repeated injections of nutrients can be applied on poor nutrient soils. Further studies should be carried out to investigate the efficacy of MICP via stimulation and augmentation in soils of varying environments, organic carbon content, microbial communities. Other pathways and bacterial isolates should also be explored and future studies should be targeted in large scale columns so that influence of microbial carbonates on soil properties can be verified from improved mechanical properties of granular materials.

## Author contributions

ND collected the soil samples, designed research, supervised work, analyzed data and wrote the manuscript. WA performed experiments and prepared figures. EW contributed in writing the manuscript and analyzing microbiological work. AM contributed in writing the manuscript. All authors read and approved the final manuscript.

### Conflict of interest statement

The authors declare that the research was conducted in the absence of any commercial or financial relationships that could be construed as a potential conflict of interest.
